# Dysbiosis and Immune Crosstalk in Experimental Diabetic Periodontitis: A Systemic Review and Meta-Analysis of Preclinical Murine Studies

**DOI:** 10.3390/ijms27125499

**Published:** 2026-06-18

**Authors:** Amani M. Harrandah

**Affiliations:** Department of Basic Oral and Clinical Sciences, College of Dental Medicine, Umm Al Qura University, Makkah 24381, Saudi Arabia; amharrandah@uqu.edu.sa

**Keywords:** oral microbiome, periodontitis, Diabetes Miletus, Interleukin-17, immune activation

## Abstract

Diabetes mellitus (DM) fundamentally disrupts the oral microbiome, initiating a dysbiotic shift that drives progressive periodontal tissue breakdown. This transition is mediated by complex, bidirectional immune crosstalk, primarily centering on the upregulation of the Th17/Interleukin-17 (IL-17) inflammatory pathway. This systematic review and meta-analysis quantified the specific impact of this diabetic microbiota on immune activation and periodontal destruction. A comprehensive search of PubMed/MEDLINE, Scopus, Web of Science, and the Cochrane Library was conducted for studies published up to 2026. Eligible studies included assessing oral/salivary microbiome shifts and their localized or systemic immunological consequences in diabetic periodontitis. A random-effects meta-analysis synthesized standardized mean differences (Hedges’ g) to evaluate the magnitude of these effects. Quantitative synthesis of preclinical data (four studies yielding eight discrete comparisons) revealed that exposure to a diabetic/dysbiotic microbiota significantly increased overall immune activation and periodontal inflammation relative to eubiotic controls (pooled Hedges’ g = 3.73, 95% CI 2.96–4.51). Subgroup analyses confirmed profound, statistically significant effects specifically on the Th17/IL-17 axis (g = 4.03) and periodontal bone destruction pathways (g = 3.37). Preclinical murine data suggests diabetes-associated oral dysbiosis may contribute to periodontal destruction by upregulating the Th17/IL-17 immune axis. However, direct extrapolation to humans is restricted, necessitating further clinical studies to validate these findings.

## 1. Introduction

The relationship between Diabetes Miletus (DM) and periodontitis is one of the most thoroughly documented bidirectional links in oral-systemic medicine. Periodontitis is widely recognized as the sixth major complication of diabetes, with epidemiological data consistently showing that diabetic patients face a significantly higher risk of developing severe periodontal destruction compared to normoglycemic individuals [1,2]. Conversely, the systemic inflammation originating from chronic periodontal infection can exacerbate insulin resistance and negatively impact glycemic control. While the physiological impact of chronic hyperglycemia—such as advanced glycation end-product (AGE) accumulation and altered neutrophil function—is well understood, recent advances in high-throughput 16S rRNA sequencing have revealed that DM fundamentally disrupts the local oral ecosystem. This metabolic disruption precipitates a critical shift in the biodiversity and composition of the oral microbiome, serving as a primary mechanism by which DM initiates and aggravates periodontal tissue breakdown [3,4].

Historically, the etiology of periodontitis was heavily attributed to the targeted virulence of specific periopathogens, most notably the ‘Red Complex’ consortium (*Porphyromonas gingivalis*, *Treponema denticola*, and *Tannerella forsythia*). Rather than being rendered obsolete by modern oral microbiology, the foundational significance of these pathogens has been mechanistically integrated into the contemporary Polymicrobial Synergy and Dysbiosis (PSD) model. The PSD model reframes the Red Complex—and specifically keystone pathogens like *P. gingivalis*—not as isolated, independent causative agents, but as critical orchestrators of community-wide dysbiosis. By subverting the host immune response, these pathogens promote a pathogenic shift in the broader microbial biofilm. It is this synergy between the dysbiotic microbial community and the resulting disproportionate, destructive host-immune interactions that drives the pathogenesis of periodontitis, a cycle that is further amplified by systemic metabolic disruptions such as diabetes [1,5]. This dysbiotic shift increases the overall pathogenicity of the oral flora. Crucially, studies utilizing murine models have demonstrated that the diabetic oral microbiome is inherently more destructive; when oral microbiota from diabetic mice is transferred to wild-type germ-free recipients, it induces significantly more periodontal inflammation and bone loss than microbiota from healthy donors [6,7]. This indicates that the microbial communities in diabetic patients are not merely pathogenic bystanders but are actively reprogrammed into a more virulent state that can independently drive disease progression.

The transition from eubiosis to dysbiosis in the diabetic periodontium is driven by a complex, bidirectional immune crosstalk that centers on the upregulation of Interleukin-17 (IL-17). Under the systemic stress of diabetes mellitus (DM), the accumulation of Advanced Glycation End-products (AGEs) and their interaction with RAGE receptors sensitize the Th17 pathway, leading to an exaggerated local expression of IL-17. This cytokine acts as a critical bridge in the Polymicrobial Synergy and Dysbiosis (PSD) model; while it directly drives osteoclastogenesis and alveolar bone resorption via the RANKL pathway, it simultaneously reshapes the subgingival environment. By promoting chronic inflammation and tissue breakdown, IL-17 provides a steady supply of heme and protein-derived nutrients that favor the selection of inflammophilic pathobionts. This immune-microbiome axis creates a self-sustaining, destructive feedback loop where host inflammatory dysfunction and microbial synergy mutually reinforce one another, locking the periodontium in a state of progressive disease [6,8,9,10].

Furthermore, the immunological consequences of this dysbiosis are not confined to the oral cavity. The oral microbiota acts as an immunological bridge capable of modulating systemic responses. For instance, exposure to a human periodontitis-associated salivary microbiome in diabetic models has been shown to trigger significant systemic immune alterations, including the upregulation of adaptive Th17 cells in the spleen and the systemic elevation of pro-inflammatory mediators such as TNF-α and IL-1β [11]. Parallel mechanisms observed in the gut–immune axis demonstrate that modulating the microbiome via probiotics or complex dietary polysaccharides can actively suppress autoimmune diabetes by promoting tolerogenic dendritic cells and regulatory T cells (Tregs) [12,13,14]. This suggests that mucosal interfaces—both oral and intestinal—serve as critical rheostats for systemic immune tolerance.

Despite the growing body of primary literature, comprehensive syntheses quantifying the specific microbial shifts and subsequent immune signaling pathways in diabetic periodontitis remain fragmented. Therefore, this systematic review and meta-analysis aim to rigorously evaluate the distinct taxonomic signatures of the dysbiotic oral microbiome under diabetic conditions. Furthermore, it seeks to elucidate the specific localized and systemic immune crosstalk mechanisms driven by this altered microbiota, highlighting potential therapeutic targets for intercepting the diabetes–periodontitis pathogenic axis.

## 2. Materials and Methods

### 2.1. Protocol and Registration

This systematic review and meta-analysis were conducted and reported in accordance with the Preferred Reporting Items for Systematic Reviews and Meta-Analyses (PRISMA) statement guidelines. The review protocol was established a priori to define the research question, search strategy, inclusion/exclusion criteria, and analytical models. The protocol was registered in the PROSPERO database under the registration number CRD420261377606.

While the primary data extraction was conducted by a single author, the search strategy and extraction protocols underwent independent external verification by a professional academic service to ensure strict compliance with PRISMA guidelines.

### 2.2. Search Strategy

A comprehensive, systematic literature search was conducted to identify relevant studies published up to January 2026. The primary electronic databases searched included PubMed/MEDLINE, Scopus, Web of Science, and the Cochrane Library. The search strategy utilized a combination of Medical Subject Headings (MeSH) and free-text keywords related to the three core domains of the review: (1) diabetes mellitus, (2) periodontitis or periodontal disease, and (3) oral microbiome or immune crosstalk.

The Boolean search string applied was as follows:

(“Diabetes Mellitus” OR “Type 1 Diabetes” OR “Type 2 Diabetes” OR “Hyperglycemia”) AND (“Periodontitis” OR “Periodontal Disease” OR “Alveolar Bone Loss”) AND (“Microbiome” OR “Microbiota” OR “Dysbiosis” OR “16S rRNA” OR “Immune Crosstalk” OR “IL-17” OR “Th17” OR “Tregs”).

Additionally, the reference lists of included articles and relevant review papers were manually screened to identify any further eligible studies.

### 2.3. Eligibility Criteria

The PICOS (Population, Intervention/Exposure, Comparison, Outcome, Study Design) framework was used to establish the following eligibility criteria:

Inclusion Criteria:

- Study Design: In vivo animal models (e.g., db/db mice, NOD mice) and human clinical studies (observational, cross-sectional, or randomized controlled trials).
- Population: Human patients diagnosed with Type 1 or Type 2 Diabetes Mellitus with concomitant periodontal disease, or murine models mimicking these conditions.
- Exposure: Evaluation of the oral/salivary microbiome (e.g., via 16S rRNA gene sequencing) and its systemic or localized immunological effects.
- Outcomes: Studies reporting quantitative data on microbial taxonomic shifts (e.g., Firmicutes, Bacteroidetes, Streptococcus, Fusobacteria) and/or local/systemic immune markers (e.g., IL-17, TNF-α, IL-1β, Th17 cell frequency, Treg differentiation, alveolar bone loss).

Exclusion Criteria:

- Studies focusing on unrelated systemic diseases (e.g., Coeliac disease) without addressing diabetes or periodontitis.
- Basic science or microbiological studies evaluating gut or intestinal homeostasis without a clear oral-systemic or diabetic context.
- Studies investigating purely mouth-to-gut microbial transfer in Type 1 Diabetes without assessing periodontal parameters or immune crosstalk.
- Review articles, editorials, conference abstracts, or case reports lacking primary, extractable quantitative data.

### 2.4. Study Selection and Data Extraction

**Figure 1:**
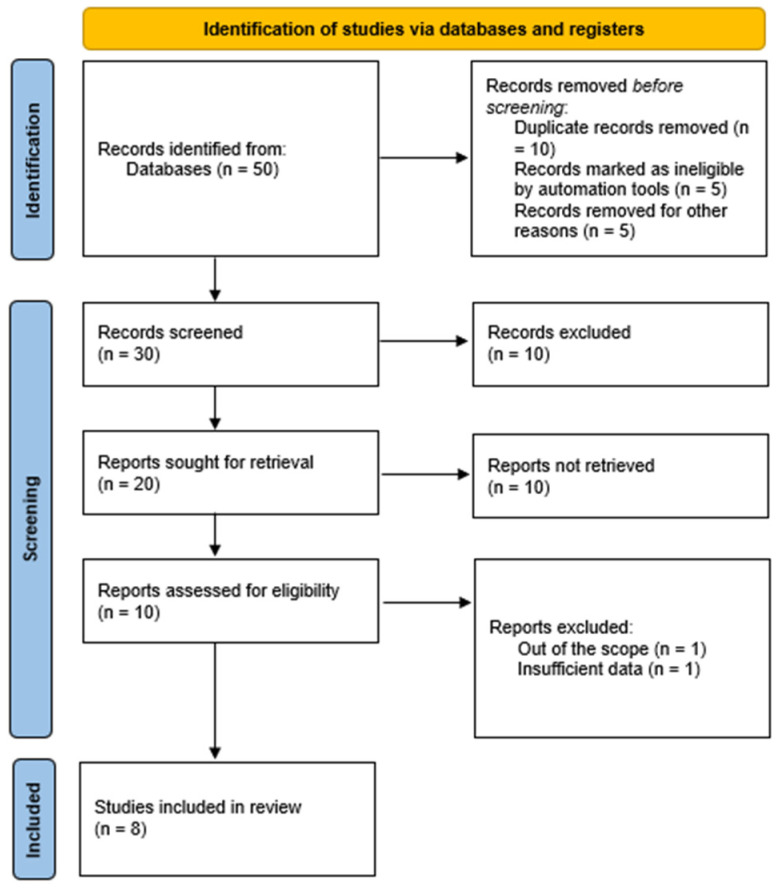
PRISMA flowchart for reports which included in the searches of database.

Titles and abstracts of all identified records were screened for relevance. Full-text articles of potentially eligible studies were then retrieved and assessed against the predefined inclusion/exclusion criteria (Figure 1).

Data from the included studies were extracted using a standardized, pre-piloted data extraction form. The extracted variables included the following:

→ Study Characteristics: First author, year of publication, study design, and country of origin.
→ Subject Demographics: Human (sample size, age, HbA1c levels, duration of diabetes) or Animal (strain, method of diabetes induction).
→ Microbiological Data: Sequencing platform, target region (e.g., V1–V2 or V3–V4 of 16S rRNA), alpha/beta diversity metrics, and significantly altered taxa.
→ Immunological Data: Key cytokines (IL-17, TNF-α, IL-1β), T-cell profiling (Th17/Treg ratios), and periodontal clinical parameters or bone loss measurements.

### 2.5. Quality Assessment and Risk of Bias

The methodological quality of the studies included was carefully assessed. For human observational studies, the Newcastle–Ottawa Scale (NOS) was utilized, evaluating cohort selection, comparability, and outcome assessment. For animal studies, the Systematic Review Centre for Laboratory animal Experimentation (SYRCLE) risk of bias tool was applied to assess selection bias, performance bias, detection bias, attrition bias, and reporting bias.

### 2.6. Statistical Analysis (Meta-Analysis)

Two prespecified objectives were evaluated: (1) the effect of diabetic microbiota on immune activation (Th17/IL-17 axis), and (2) its effect on periodontal inflammation and bone destruction. Due to the nature of continuous outcomes measured on different biological scales, all results were synthesized using standardized mean differences (SMDs) to enable comparability across studies. The SMD expresses the difference between experimental and control groups relative to within-study variability [15,16]. Effect sizes were calculated as Hedges’ g, a small-sample–corrected version of Cohen’s d that reduces positive bias in effect estimation when sample sizes are limited, which is particularly relevant for preclinical animal studies [17]. For each comparison, Hedges’ g, its variance, standard error, and 95% confidence intervals were derived from reported group means, dispersion measures, and sample sizes using established formulas [15]. In the primary analysis, reported dispersion measures were treated as standard deviations (SDs) when calculating Hedges’ g. Because some included studies did not consistently distinguish whether the reported dispersion measure represented an SD or a standard error (SE), we conducted a conservative sensitivity analysis in which potentially SE-based values were converted to SDs using SD = SE × √n before recalculating effect sizes. This sensitivity analysis was used to evaluate whether the pooled estimate was robust to uncertainty in dispersion reporting. Because several studies reported multiple related outcomes, effect sizes were first aggregated at the study level using inverse-variance weighting to obtain a single independent estimate per study before conducting the overall meta-analysis [15]. A random-effects meta-analysis was specified a priori to account for expected biological and methodological variability across animal models, including differences in microbiota transfer, tissue compartments, and outcome measurements. The between-study variance (τ^2^) was estimated using the Paule–Mandel with Hartung–Knapp adjustment [18]. In the present dataset, τ^2^ converged to zero, resulting in equivalence between random- and fixed-effect estimates. Statistical heterogeneity was assessed using Cochran’s Q statistic, its associated *p* value, and the I^2^ statistic, which quantifies the proportion of total variability attributable to between-study heterogeneity rather than sampling error [19]. Given the small number of included studies, heterogeneity estimates were interpreted cautiously, recognizing the limited statistical power to detect true between-study variability in small meta-analyses [20].

Prespecified subgroup analyses were conducted using a χ^2^ test for subgroup differences (Cochran’s Q), with statistical significance defined as *p* < 0.05 according to biological domain (immune activation vs. periodontal inflammation) to evaluate domain-specific effects. To assess robustness, a leave-one-out sensitivity analysis at the study level was performed by sequentially omitting each effect size and recalculating the pooled estimate [21]. Potential small-study effects were explored using Egger’s regression test, which evaluates the association between effect size magnitude and its standard error [22]. However, because fewer than ten effect sizes were included, this analysis was considered exploratory and interpreted with caution. All statistical tests were two-sided. Statistical significance was defined as a 95% confidence interval that did not cross zero. All analyses were conducted using SAS version 9.4 (SAS Institute Inc., Cary, NC, USA) and implemented using validated statistical procedures to ensure reproducibility.

## 3. Results

### 3.1. Study Selection and Characteristics

The systematic search and screening process yielded a core group of studies suitable for qualitative and quantitative synthesis. To manage methodological heterogeneity, these were stratified into two primary domains: (1) observational human clinical trials evaluating taxonomic shifts in the oral microbiome, and (2) in vivo murine models investigating immunological crosstalk. For the quantitative meta-analysis, four independent in vivo preclinical studies were included, comprising a total of eight discrete comparisons. These comparisons evaluated the impact of a diabetic/dysbiotic microbiota on two predefined biological domains: immune activation (specifically the Th17/IL-17 axis) and periodontal inflammation with bone destruction.

### 3.2. Primary Meta-Analysis

All eight comparisons demonstrated positive effect sizes, consistently indicating increased immune activation and periodontal inflammation in groups exposed to the diabetic/dysbiotic microbiota compared to normoglycemic or eubiotic controls. Standardized mean differences (Hedges’ g) at the individual comparison level ranged from 2.06 to 4.54.

**Figure 2:**
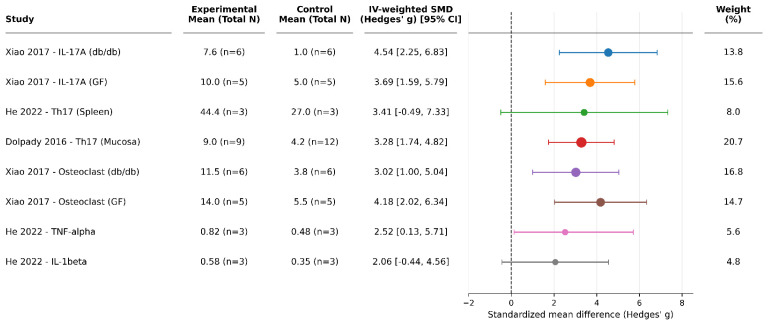
Inverse-variance-weighted forest plot of standardized mean differences (Hedges’ g) comparing diabetic versus control microbiota [6,11,12].

**Figure 3:**
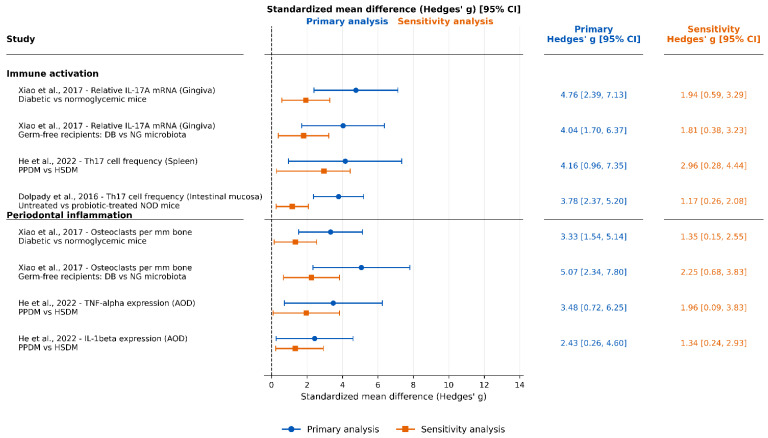
Forest plot of standardized mean differences (Hedges’ g) for immune activation and periodontal outcomes associated with diabetic microbiota (random-effects model) [6,11,12].

In the primary overall analysis, utilizing a random-effects model where dispersion was treated as standard deviation, the pooled estimate revealed a massive and statistically significant effect size (Hedges’ g = 3.73, 95% CI 2.96–4.51) (Figure 2). Notably, no statistical heterogeneity was detected across the included comparisons (Q = 3.42, df = 7, *p* = 0.84; τ^2^ = 0; I^2^ = 0%) (Table 1, Figure 3).

### 3.3. Subgroup Analyses by Biological Domain

To further delineate the effects, subgroup analyses were conducted for the two predefined biological domains (Table 2).

- **Immune Activation (Th17/IL-17 Axis): **

The pooled effect for immune activation outcomes remained exceptionally large and significant (g = 4.03, 95% CI 2.98–5.07). Within this domain, individual effect sizes were substantial for both IL-17A expression (g = 4.76, 95% CI 2.39–7.13; and g = 4.04, 95% CI 1.70–6.37) and Th17 cell frequency across different compartments (g = 4.16, 95% CI 0.96–7.35; and g = 3.78, 95% CI 2.37–5.20). No within-group heterogeneity was observed (Q = 0.52, df = 3, *p* = 0.91; I^2^ = 0%).

Four comparisons evaluated immune activation outcomes, including IL-17A mRNA expression in gingival tissue and Th17-cell frequency in systemic and mucosal immune compartments. In the primary analysis, all studies demonstrated large positive effect sizes favoring diabetes-associated microbiota exposure or untreated diabetic conditions relative to controls, with Hedges’ g values ranging from 3.78 to 4.76. The largest effect was observed for IL-17A mRNA expression in diabetic versus normoglycemic mice (Hedges’ g = 4.76, 95% CI: 2.39–7.13), while the smallest effect was observed for Th17-cell frequency in untreated versus probiotic-treated mice (Hedges’ g = 3.78, 95% CI: 2.37–5.20). Similar directional associations were observed in germ-free microbiota-transfer models and salivary microbiome transplantation experiments, indicating consistently increased immune activation associated with diabetes-related oral microbial dysbiosis.

Sensitivity analyses demonstrated attenuation of effect estimates but preserved the overall direction of association across all comparisons. Sensitivity Hedges’ g values ranged from 1.17 to 2.96, suggesting that although effect magnitudes were reduced under conservative assumptions, diabetes-associated microbial exposure remained consistently associated with elevated IL-17A expression and increased Th17-cell activity.

- **Periodontal Inflammation and Bone Destruction:**

The pooled effect size for periodontal outcomes was similarly robust (g = 3.37, 95% CI 2.21–4.53). This was driven by significant increases in osteoclast activity (g = 3.33, 95% CI 1.51–5.14; and g = 5.07, 95% CI 2.34–7.80), as well as the expression of pro-inflammatory cytokines TNF-α (g = 3.48, 95% CI 0.72–6.25) and IL-1β (g = 2.43, 95% CI 0.26–4.60). Consistent with the immune domain, no statistical heterogeneity was detected (Q = 2.22, df = 3, *p* = 0.53; I^2^ = 0%).

Four comparisons evaluated periodontal inflammatory outcomes, including osteoclast activity, TNF-α expression, and IL-1β expression. In the primary analysis, all studies demonstrated large positive effect sizes, with Hedges’ g values ranging from 2.43 to 5.07. The strongest association was observed in the germ-free microbiota-transfer model evaluating osteoclast activity (Hedges’ g = 5.07, 95% CI: 2.34–7.80), whereas the smallest effect was observed for IL-1β expression (Hedges’ g = 2.43, 95% CI: 0.26–4.60). Osteoclast-related outcomes consistently showed the largest effect estimates across studies, supporting a strong association between diabetes-associated oral microbiota and alveolar bone resorption pathways. Sensitivity analyses yielded lower but directionally consistent effect estimates, ranging from 1.34 to 2.25. Although confidence intervals widened in several comparisons, positive associations remained evident for osteoclast activity, TNF-α expression, and IL-1β expression, indicating robustness of the observed relationship between diabetes-associated microbial dysbiosis and periodontal inflammatory responses.

### 3.4. Sensitivity Analysis and Robustness

Sensitivity analyses were performed to evaluate the stability of the primary findings. When alternative variance assumptions were applied (converting Standard Error to Standard Deviation), the pooled estimate yielded a lower, yet still statistically significant, effect size (g = 1.61, 95% CI 1.13–2.09). This attenuation was expected because converting SEs to SDs increases the dispersion estimate used in the denominator of the standardized mean difference, thereby reducing the magnitude of Hedges’ g. Thus, the sensitivity analysis indicates that the magnitude of the pooled effect was sensitive to assumptions regarding dispersion reporting. However, the direction of association and statistical significance were preserved, supporting the robustness of the biological conclusion while indicating that the exact effect magnitude should be interpreted cautiously. This indicates that while the magnitude of the effect is sensitive to variance specification, the overall direction and significance of the effect are robust.

Additionally, leave-one-out sensitivity analyses demonstrated that no single comparison exerted a disproportionate influence on the overall results. The pooled estimates remained highly stable across all iterations (ranging from g = 3.58 to 3.91), and statistical heterogeneity consistently remained at I^2^ = 0%.

### 3.5. Publication Bias and Small-Study Effects

**Figure 4:**
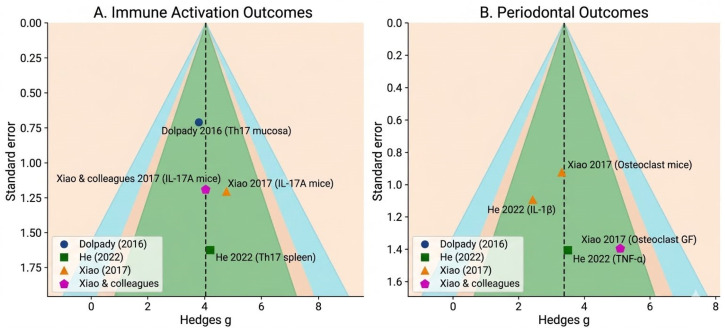
Funnel plots of Hedges’ g versus standard error for evaluation of small-study effects. (**A**) Immune activation outcomes (Th17/IL-17 axis). (**B**) Periodontal inflammation and bone destruction outcomes. Vertical lines indicate pooled effect estimates; dashed lines represent pseudo 95% confidence limits. Visual inspection suggests no clear asymmetry, no obvious pattern indicating small-study effects or publication bias although interpretation is limited by the small number of included studies [6,11,12].

Exploratory assessments for small-study effects and publication bias were conducted. Visual inspection of the funnel plots for both immune activation and periodontal outcomes (Figure 4) revealed a symmetrical distribution of effect sizes around the pooled estimates, with no clear asymmetry or clustering. This visual assessment was supported quantitatively by Egger’s regression test, which found no statistically significant evidence of asymmetry (intercept = 0.99, SE = 1.11, *p* = 0.38). However, it must be noted that these assessments are underpowered due to the limited number of comparisons (k = 8) available for analysis.

## 4. Discussion

This meta-analysis demonstrates a substantial and consistent association between the diabetic microbiota and both heightened immune activation and severe periodontal tissue destruction in preclinical models. These findings strongly support a biologically coherent link wherein diabetes-associated microbial dysbiosis acts as a primary driver of Th17-mediated inflammatory and osteoclastogenic pathways.

The bidirectional relationship between diabetes and periodontitis creates a “vicious cycle” where chronic hyperglycemia fuels a dysbiotic oral microbiome, which in turn sustains systemic inflammation and impairs glycemic control [23]. This systemic overstimulation triggers a cascade of oxidative stress and inflammatory signaling that directly impairs periodontal healing. Within the gingival tissues, the AGE-RAGE axis amplifies the production of matrix metalloproteinases (MMPs) and pro-inflammatory mediators, leading to the rapid breakdown of collagen and the periodontal ligament. Furthermore, this axis significantly inhibits osteoblast function and promotes osteoclast survival, tilting the balance toward the aggressive alveolar bone loss characteristic of diabetic periodontitis [9,24,25,26,27]. Concurrently, elevated blood glucose levels inhibit essential innate immune functions—specifically reducing neutrophil migration, degranulation, and phagocytosis—which decreases microbial clearance and allows pathogenic bacteria to thrive. Consequently, this dysregulated host response fosters a highly virulent oral microbiota that aggressively accelerates periodontal bone loss [28,29].

From a statistical perspective, the observed absence of statistical heterogeneity (I^2^ = 0%) should not be misinterpreted as true biological homogeneity. The small number of included studies substantially limits the statistical power required to accurately detect underlying between-study variability. Furthermore, the unusually large effect sizes (Hedges’ g ≈ 3–5) are likely a byproduct of the highly controlled nature of preclinical in vivo models. These experimental designs typically utilize specific pathogen-free housing, standardized microbial inoculations, and identical diets, which—when combined with small sample sizes and strong experimental group contrasts—can artificially inflate effect magnitudes. To verify the stability of these results, sensitivity analyses were conducted. While these analyses demonstrated some attenuation of effect sizes under alternative variance assumptions, the overall direction and statistical significance were consistently preserved. Additionally, leave-one-out analyses confirmed that the overarching conclusions were robust and not disproportionately driven by any single outlier study.

While our meta-analysis demonstrated massive effect sizes (e.g., *g* = 4.03 and 5.07) regarding immune expression and alveolar bone loss, these quantitative findings must be critically interpreted within the context of preclinical design. Such extreme magnitudes are largely an artifact of the methodology rather than a direct reflection of human clinical reality. Murine models utilize genetically homogeneous, highly inbred strains housed in strictly controlled specific-pathogen-free (SPF) environments with uniform diets, effectively eliminating the genetic, environmental, and behavioral heterogeneity that dilutes effect sizes in human cohorts.

Furthermore, these inflated metrics underscore a fundamental biological translation problem. The native murine oral microbiome differs substantially from that of humans; notably, mice do not naturally harbor keystone human periopathogens, such as the Red Complex species. Consequently, experimental periodontitis in these models relies on artificial inoculation or mechanical ligation. These interventions induce an acute, rapid burst of localized inflammation and microbial shifting that contrasts sharply with the chronic, decades-long dysbiotic progression of human diabetic periodontitis. Therefore, while the massive effect sizes confirm the *mechanistic presence* of a diabetic-periodontal link, they do not accurately represent the *clinical magnitude* expected in human patients.

A critical finding of this analysis is the exacerbation of the Th17/IL-17 immune response in the diabetic models. Importantly, this hyper-inflammatory axis does not merely inflict collateral tissue damage; it actively drives and sustains microbial dysbiosis. According to the inflammophilic hypothesis, the overproduction of IL-17 and the subsequent massive recruitment of neutrophils create a highly inflamed microenvironment. This hyper-inflammation increases the flow of nutrient-rich gingival crevicular fluid, releasing tissue breakdown products such as host-derived heme and degraded collagen peptides.

These specific inflammatory byproducts serve as primary nutrient sources for proteolytic, dysbiotic pathogens like *P. gingivalis*, fundamentally altering the ecological niche of the oral cavity. Consequently, the diabetic amplification of the Th17/IL-17 axis establishes a pathogenic positive feedback loop: the altered host immune response actively cultivates a more pathogenic, inflammophilic microbiome, which in turn perpetually triggers further immune-mediated alveolar bone resorption.

Overall, these results provide compelling experimental evidence that diabetes induces a dysbiotic microbiota, which in turn drives profound immune and periodontal dysregulation. However, the limited number of available studies and the inherent methodological diversity of the models constrain the precision and broader clinical generalizability of these findings. Larger, rigorously standardized clinical and preclinical studies are imperative to fully validate these mechanistic pathways and to more accurately quantify true heterogeneity.

## 5. Conclusions

In conclusion, this systematic review and meta-analysis of preclinical murine models suggests that diabetes mellitus is associated with a dysbiotic shift in the oral microbiome, which may contribute to progressive periodontal tissue destruction. Our findings indicate that exposure to this diabetes-associated microbiota is linked to increased localized and systemic immune activation, particularly involving the upregulation of the Th17/IL-17 inflammatory axis and enhanced osteoclastogenesis. These results support the hypothesis that the altered microbial community actively participates in the bidirectional relationship between diabetes and periodontitis.

However, the substantial effect sizes observed are likely amplified by the highly controlled nature and specific-pathogen-free environments of the limited number of available in vivo studies, which restricts direct extrapolation to human clinical pathology. Consequently, while these preclinical data provide valuable mechanistic insights into the immune-microbiome axis, definitive conclusions regarding human disease pathogenesis cannot yet be drawn. Future research must prioritize rigorously standardized, large-scale clinical and translational studies to determine if these complex host–microbiome interactions—and their potential as therapeutic targets—are applicable in human populations.

## Figures and Tables

**Table 1:** Random-effects meta-analysis of the effect of diabetic microbiota on immune activation and periodontal inflammation.

Domain	Study	Study Design/Model	Experimental Exposure/Intervention	Comparator	Biological Compartment/Sample	Extracted Outcome	Comparison Used for Meta-Analysis	Hedges’ g (Primary) ^1^	Weight (%) ^2^	95% CI	Hedges’ g (Sensitivity) ^1^	95% CI
Immune activation	Xiao et al., 2017 [6]	In vivo murine diabetic periodontitis model	Diabetes-associated oral microbiota/diabetic condition	Normoglycemic mice	Gingival tissue	IL-17A mRNA expression	Diabetic vs. normoglycemic mice	4.76	13.8	2.39–7.13	1.94	0.59–3.29
Immune activation	Xiao et al., 2017 [6]	Germ-free microbiota-transfer mouse model	Oral microbiota transferred from diabetic mice	Oral microbiota transferred from normoglycemic mice	Gingival tissue/periodontal tissue	IL-17A mRNA expression	Germ-free recipients: diabetic microbiota vs. normoglycemic microbiota	4.04	15.6	1.70–6.37	1.81	0.38–3.23
Immune activation	He et al., 2022 [11]	Diabetic mouse model receiving human salivary microbiome transplantation	Human periodontitis-associated salivary microbiome transplanted into diabetic mice	Healthy-saliva microbiome transplanted into diabetic mice	Spleen/systemic immune compartment	Th17 cell frequency	PPDM vs. HSDM	4.16	8.0	0.96–7.35	2.36	0.28–4.44
Immune activation	Dolpady et al., 2016 [12]	Non-obese diabetic mouse model/oral probiotic intervention	Untreated autoimmune diabetes model	Oral probiotic-treated model	Mucosal immune compartment	Th17 cell frequency	Untreated vs. probiotic-treated	3.78	20.7	2.37–5.20	1.17	0.26–2.08
Periodontal inflammation	Xiao et al., 2017 [6]	In vivo murine diabetic periodontitis model	Diabetes-associated oral microbiota/diabetic condition	Normoglycemic mice	Periodontal tissue/alveolar bone compartment	Osteoclast activity	Diabetic vs. normoglycemic mice	3.33	16.8	1.51–5.14	1.35	0.15–2.55
Periodontal inflammation	Xiao et al., 2017 [6]	Germ-free microbiota-transfer mouse model	Oral microbiota transferred from diabetic mice	Oral microbiota transferred from normoglycemic mice	Periodontal tissue/alveolar bone compartment	Osteoclast activity	Germ-free recipients: diabetic microbiota vs. normoglycemic microbiota	5.07	14.7	2.34–7.80	2.25	0.68–3.83
Periodontal inflammation	He et al., 2022 [11]	Diabetic mouse model receiving human salivary microbiome transplantation	Human periodontitis-associated salivary microbiome transplanted into diabetic mice	Healthy-saliva microbiome transplanted into diabetic mice	Alveolar/periodontal outcome domain	TNF-α expression	PPDM vs. HSDM	3.48	5.6	0.72–6.25	1.96	0.09–3.83
Periodontal inflammation	He et al., 2022 [11]	Diabetic mouse model receiving human salivary microbiome transplantation	Human periodontitis-associated salivary microbiome transplanted into diabetic mice	Healthy-saliva microbiome transplanted into diabetic mice	Alveolar/periodontal outcome domain	IL-1β expression	PPDM vs. HSDM	2.43	4.8	0.26–4.60	1.34	−0.24–2.93

^1^ Hedges’ g (Primary) represents the standardized mean difference calculated using reported dispersion measures treated as standard deviations (SDs). Hedges’ g (Sensitivity) reflects effect sizes recalculated after converting dispersion measures assumed to be standard errors (SEs) into SDs using SD = SE × √n. Effect sizes are presented with 95% confidence intervals. Weights (%) were derived from inverse-variance random-effects models incorporating between-study variance (τ^2^). ^2^ Weight represents the relative contribution of each extracted comparison to the displayed comparison-level synthesis. In the revised primary analysis, where multiple related outcomes were reported from the same study, comparison-level effect sizes should be aggregated at the study level within the biological domain before estimating pooled effects, or alternatively modeled using a multilevel/three-level meta-analysis to account for within-study dependence. Abbreviations: HSDM, healthy salivary microbiome-treated diabetic mice; IL-1β, interleukin-1 beta; IL-17A, interleukin-17A; PPDM, periodontitis-associated salivary microbiome-treated diabetic mice; Th17, T helper 17 cells; TNF-α, tumor necrosis factor alpha.

**Table 2:** Pooled meta-analysis results for immune activation and periodontal inflammation outcomes associated with diabetic microbiota.

Analysis	Domain	k	Hedges’ g (95% CI)	SE	Q	df	*p* (Q)	τ^2^	I^2^ (%)
Overall (primary model)	All outcomes	8	3.73 (2.96–4.51)	0.39	3.42	7	0.84	0.00	0
Subgroup analysis	Immune activation	4	4.03 (2.98–5.07)	0.53	0.52	3	0.91	0.00	0
	Periodontal inflammation	4	3.37 (2.21–4.53)	0.59	2.22	3	0.53	0.00	0
Sensitivity analysis	All outcomes (SE → SD)	8	1.61 (1.13–2.09)	0.25	2.77	7	0.91	0.00	0
Publication bias	Egger intercept	—	0.99	1.11	—	—	0.38	—	—

k denotes the number of effect sizes included in each analysis. Effect sizes are reported as Hedges’ g (standardized mean difference) with 95% confidence intervals. SE indicates the standard error of the pooled estimate. Q represents Cochran’s heterogeneity statistic with corresponding degrees of freedom (df) and *p* value (*p* [Q]). τ^2^ denotes between-study variance, and I^2^ represents the proportion of total variability attributable to between-study heterogeneity. The primary analysis assumes reported dispersion measures represent standard deviations (SD). In the sensitivity analysis, dispersion measures assumed to represent standard errors (SE) were converted to SD prior to effect size estimation. Egger’s regression test was performed to assess small-study effects; results should be interpreted cautiously given the limited number of included effect sizes (k < 10).

## Data Availability

No new data were created or analyzed in this study. Data sharing is not applicable to this article.

## Abbreviations

The following abbreviations are used in this manuscript:

AGE	Advanced Glycation End-product
DM	Diabetes Mellitus
IL-17	Interleukin-17
HSDM	High-Sucrose Diet Model
PPDM	Polymicrobial Periodontitis Diabetic Model
IL-1β	Interleukin-1 beta
MeSH	Medical Subject Headings
NOD	Non-Obese Diabetic (in reference to murine models)
PICOS	Population, Intervention/Exposure, Comparison, Outcome, Study Design
PRISMA	Preferred Reporting Items for Systematic Reviews and Meta-Analyses
PSD	Polymicrobial Synergy and Dysbiosis
RAGE	Receptor for Advanced Glycation End-products
RANKL	Receptor Activator of Nuclear Factor Kappa B Ligand
rRNA	Ribosomal Ribonucleic Acid (as in 16S rRNA)
Th17	T helper 17 cells
TNF-α	Tumor Necrosis Factor-alpha
Tregs	Regulatory T cells
